# Factors associated with deficiencies in packaging of surgical instrument by staff at a single center in China

**DOI:** 10.1186/s12913-022-08030-1

**Published:** 2022-05-17

**Authors:** Yanhua Chen, Liangying Yi, Juan Hu, Ruixue Hu

**Affiliations:** 1 Department of Sterile Processing Nursing, West China Second University Hospital, Sichuan University/West China School of Nursing, Sichuan University, Chengdu, Sichuan China; 2 Key Laboratory of Birth Defects and Related Diseases of Women and Children (Sichuan University), Ministry of Education, Chengdu, Sichuan China

**Keywords:** Accident prevention, Equipment failure, Factor analysis, Product packaging, Surgical instruments

## Abstract

**Background:**

Surgical instrument packaging quality directly affects the safety and performance of surgery. We aimed to investigate the factors causing defects in surgical instrument packaging and recommend strategies to prevent defects in surgical instrument packaging.

**Methods:**

We collected surgical instrument packaging data regarding age, gender, length of service, educational background, number of staff dealing with packaging, time period of packaging, instrument specification, where the wrap was intact, whether the wrap reached the required quality of cleaning, and whether the instruments were satisfactorily cleaned in compliance with guidelines from 5000 surgical instrument packages during June-December 2018 at Central Sterile Supply Department of the West China Second University Hospital, Sichuan University. Meanwhile, a questionnaire survey completed by the operating room staff using surgical instruments was used to measure the level of their satisfaction with the instruments in the packages. We utilized single-factor analysis to investigate possible factors that might cause defects in surgical instrument packaging, and conducted multivariate logistic regression analysis of the factors associated with defects in packaging.

**Results:**

Length of service, educational background, number of staff dealing with packaging, time period of packaging, instrument structure, whether the wrap was intact, whether the wrap reached the required quality of cleaning, and whether the surgical instruments were satisfactorily cleaned in compliance with guidelines were the factors significantly (*P* < 0.05) associated with defects in surgical instrument packaging.

**Conclusion:**

This study reveals that various factors are associated with defects in surgical instrument packaging. Recommendations for reducing incidences of defects include improved scheduling of packaging workload, greater provision of training in packaging skills, and standardization of packaging procedure.

## Background

Due to the continuous development of surgical technology in recent years, there now exists an enormous variety of surgical instruments with complex, specialized, and precise structural design [1]. Most of the surgical instruments used in hospitals in China are reusable. Under the Management Standard of the Central Sterile Supply Department (CSSD) regulations issued by the Ministry of Health, the People’s Republic of China, surgical instruments shall be delivered to CSSD after use. CSSD staff members deal with a large number of the instruments every day. If surgical instruments are not cleaned thoroughly or if the packaging is defective, patients face the risk of surgical site infection and medical incidents [2]. The CSSD therefore plays a significant role in hospital infection control. Surgical instruments come into contact with patients’ tissues and body fluids. Surgical instrument packaging entails using various packaging materials to package the reusable surgical instruments to create a sterile barrier. It involves assembly, wrapping, sealing, and labeling. Packaging is required for reusable surgical instruments, and surgical instrument packaging quality directly affects the safety and performance of surgery [3]. Previous studies have shown that the packaging defect rate of sterile packages was 1.43‰ to 1.67‰ [4–6]. In order to identify areas where the quality of nursing care should be improved and ensure patient safety, we investigated the factors causing defects in surgical instrument packaging. From our findings, we prescribe strategies for preventing defects in surgical instrument packaging.

## Methods

### Ethics

All methods were performed in accordance with the relevant guidelines and regulations. This study was performed in accordance with the Declaration of Helsinki. Ethics approval of this study was obtained from the Medical Ethics Committee of West China Second University Hospital, Sichuan University (No.: YXKY2021LSP(067)).

### Study setting

This was a descriptive research project. A total of 5000 surgical instrument packages were selected by the quality controllers from approximately 120000 packages to be sterilized at the CSSD of the West China Second University Hospital, Sichuan University during June - December 2018. The packages were sampled with a computer generated random number table. Inclusion criteria: only packages which have been assembled, wrapped, sealed and labeled, and awaiting sterilization, were selected. Exclusion criteria: overweight or oversized packages waiting to be sterilized were omitted from this study.

### Data collection

The packaging data included the following: the name of the package, category of defect, time period of packaging, staff details (names, gender, age, length of service, educational background, total number of staff dealing with packaging), instrument specification, wrapping cleaning compliance rate (percentage of the sample wraps that reached the required quality of cleaning), wrapping perfection rate (percentage of the sample wraps without damage), and instrument cleaning compliance rate (percentage of the sample instruments satisfactorily cleaned in compliance with guidelines).

### Surgical instrument packaging quality evaluation

Surgical instrument packaging quality evaluation was conducted on the basis of packaging defects and satisfaction scores.

Packaging defects were detected by the quality controllers using visual inspection, magnifying glass, adenosine triphosphate fluorescence detection method, and white pull-through cords according to the the surgical instrument packaging regulations stated in the CSSD guidelines issued by the Ministry of Health of China. The defect detection process and data were doubled-checked by a second quality controller according to the same guidelines to ensure the accuracy of the data.

Packaging was considered defective for any of the following reasons: (1) the number of instruments in the package was incorrect; (2) the packaging did not achieve the desired quality in cleanliness; (3) the instrument did not meet the necessary functional demand; (4) the assembly, arrangement, or placement of instruments was incorrect; (5) accessories or labeling was wrong; or, (6) a chemical indicator was missing.

The Surgical Instrument Packaging Quality Satisfaction Survey completed by the operating room staff using surgical instruments at our hospital was used to measure the level of their satisfaction with the instruments in the packages in terms of supply (number of instruments in package), cleanliness, function, assembly, and labeling of the instruments. The survey questionnaire consisted of 10 close-ended questions. There were no open-ended questions. Each of the 10 questions had binary options: “unsatisfactory”, and “satisfactory”. A score of 0 was assigned to the response “unsatisfactory”, and a score of 1 was assigned to the response “satisfactory”. Therefore, the maximum total score of the questionnaire was 10. When a packaging defect was identified or the satisfaction score was 9 or below, the packaging was classified into the “inefficient” group. When packaging was in compliance with packaging criteria or the satisfaction score was 10, the packaging was classified into the “efficient” group.

### Statistical methods

SPSS version 20.0 was used to analyze and process the data in this study. Enumeration data and measurement data were described with [n (%)] and (μ ± s), respectively. In the single-factor analysis, continuous numerical variables were subject to normal distribution tests followed by t-tests; the Chi-square (χ^2^) test was performed on the categorical variables. Binary logistic regression was utilized to analyze the factors associated with defects in packaging. A statistically significant difference was indicated by α = 0.05, *P* < 0.05.

## Results

Defects in surgical instrument packaging were classified and statistically analyzed for all 5000 surgical instrument packages assessed. There were 103 packages with defects (2.06%), among which 24 (0.48%) contained an incorrect number of instruments, 21 (0.42%) did not achieve the desired quality in cleaning, 17 (0.34%) had functional defects, 13 (0.26%) were assembled missing chemical indicators, 12 (0.24%) had incorrect packaging labels, 8 (0.16%) had wrong packaging materials, 7 (0.14%) had wrong instrument specifications, and 1 (0.02%) had defective sealing.

### Single-factor analysis of possible factors associated with defects in packaging

There were 4897 cases (97.94%) in the efficient group and 103 cases (2.06%) in the inefficient group. Compared with the efficient group, the inefficient group had statistically significant differences (*P* < 0.05) among the factors (short time of service (< 3 years) or low levels of education among staff, high frequency of packaging in the afternoon, insufficient number of staff dealing with packaging (≤ 2 persons), and complex instrument structural design). In addition, the wrapping cleaning compliance rate, wrapping perfection rate, and instrument cleaning compliance rate among the cases in the inefficient group were lower than those of the efficient group, a difference which proved to be statistically significant (*P* < 0.05). However, between the two groups there was no statistically significant difference for staff age, staff gender, or instrument weight (*P* > 0.05), as shown in Table 1.

**Table 1:** Single-factor analysis of possible factors associated with defects in packaging

Clinical information	Inefficient group (*n* = 103)	Efficient group (*n* = 4897)	χ^2^/*t*	*P* value
Age				
≤30^a^ years old	35 (33.98)^b^	1562 (31.90)	0.201^c^	0.654
>30 years old	68 (66.02)	3335 (68.10)		
Length of service				
≤3 years	78 (75.73)	1398 (28.55)	107.928	0.000
>3 years	25 (24.27)	3499 (71.45)		
Gender				
Male	27 (26.21)	1331 (27.18)	0.048	0.827
Female	76 (73.79)	3566 (72.82)		
Educational background				
Two or three years’ higher education diploma	67 (65.05)	1511 (30.86)	54.604	0.000
Undergraduate and above	36 (34.95)	3386 (69.14)		
Time period of packaging				
Morning	44 (42.72)	2579 (52.66)	4.002	0.045
Afternoon	59 (57.28)	2318 (47.34)		
Number of staff dealing with packaging				
≤2	65 (63.11)	2501 (51.07)	5.848	0.016
>2	38 (36.89)	2396 (48.93)		
Instrument structure				
Complex	54 (52.43)	1172 (23.93)	44.254	0.000
Simple	49 (47.57)	3725 (76.07)		
Instrument weight				
Heavy	22 (21.36)	962 (19.64)	0.188	0.665
Light	81 (78.64)	3935 (80.36)		
Wrapping perfection rate (%)	84.06±2.65^d^	98.48±1.71	96.476^e^	0.000
Wrapping cleaning compliance rate (%)	84.57±3.27	98.01±1.42	103.312	0.000
Instrument cleaning compliance rate (%)	86.03±2.83	99.34±1.67	99.685	0.000

^a^Median age is 30 years

^b^n (%)

^c^Chi-square test

^d^‾x±s

^e^t-test

### Multivariate logistic regression analysis of factors associated with defects in packaging

The statistically significant influencing factors (length of service and educational background of staff, time period of packaging, number of staff dealing with packaging, instrument structure, wrapping perfection rate, wrapping cleaning compliance rate, and instrument cleaning compliance rate) in the single-factor analysis were considered as the independent variables. The occurrence of surgical instrument packaging defect (1 = yes, 0 = no) was considered as the dependant variable. Assignments of the influencing factors are shown in Table 2. The logistic regression analysis results show that surgical instrument packaging defects can be attributed to length of service, educational background, time period of packaging, number of staff dealing with packaging, instrument structure, whether the wrap was intact, whether the wrap reached the required quality of cleaning, and Whether the instruments were satisfactorily cleaned in compliance with guidelines (*P* < 0.05), as shown in Table 3.

**Table 2:** Factors associated with defects in packaging and their assignments

Factor	Code	Assignment description
Length of service	X1	1 = ≤3, 0 =>3
Educational background	X2	1 = Low, 0 = High
Time period of packaging	X3	1 = Afternoon, 0 = Morning
Number of staff dealing with packaging	X4	1 = ≤2, 0 =>2
Instrument structure	X5	1 = Complex, 0= Simple
Whether the wrap was intact	X6	1 = Yes, 0 = No
Whether the wrap reached the required quality of cleaning	X7	1 = Yes, 0 = No
Whether the instruments were satisfactorily cleaned in compliance with guidelines	X8	1 = Yes, 0 = No

**Table 3:** Multivariate logistic regression analysis of factors associated with defects in packaging

Variable	β	SE	Waldχ^2^	*P* value	OR (95% CI)
Length of service	3.692	1.195	9.544	0.002	40.141 (3.857,417.831)
Educational background	2.458	0.987	6.199	0.013	11.687 (1.687,80.951)
Time period of packaging	2.442	1.818	5.225	0.022	11.495 (1.013,130.494)
Number of staff dealing with packaging	2.192	1.015	4.667	0.031	8.955 (1.225,65.441)
Instrument structure	1.836	0.824	4.970	0.026	6.271 (1.248,31.498)
Whether the wrap was intact	2.507	0.957	6.865	0.009	12.263 (1.880,79.975)
Whether the wrap reached the required quality of cleaning	1.045	0.963	6.977	0.008	2.844 (1.569,14.204)
Whether the instruments were satisfactorily cleaned in compliance with guidelines	2.482	0.967	6.587	0.010	11.970 (1.798,79.694)

β- estimated regression coefficient for variables, *SE* sensitivity, Waldχ^2^ - chi-square value. OR = exp (β), refers to the odds ratio of a variable in the presence of influencing surgical instrument packaging quality when values of other variables are fixed. 95% CI – 95% confidence interval

## Discussion

Surgical instrument packaging is an important part in the recycling and reuse of medical instruments. The surgical instrument packaging quality directly influences the final quality of sterile items, quality of medical care, and patient safety [7]. Common surgical instrument packaging defects included insufficient number of instruments in the package, worn-out instrument with poor performance, inconsistency between labeling outside the package and instruments inside the package, unclear or altered marking outside the package, incorrect date, wrong instrument specification, wrong packaging materials, missing internal chemical indicator, and stains or holes in the cloth wrapping material. Most of these defects may be attributed to human factors such as mental fatigue, lack of energy, lack of concentration [8], heavy workload, lack of strict and standardized training, lack of rigorous work attitude, or insufficient number of staff dealing with packaging. Staff members with long work experience generally had a better understanding of packaging quality criteria.

The results of this study show that the possible factors contributed to the occurrence of surgical instrument packaging defects are as follows: (1) *Length of service*. Our study shows that the packaging staff who had served for fewer than 3 years had a higher defect rate in packaging compared with those who had served for more than 3 years, which is consistent with the result of Pan et al. [9] who pointed out that staff with insufficient work experience tend to lack vocational skills and may not foresee the potential hazards to surgery caused by surgical instrument packaging defects. (2) *Educational background and number of staff dealing with packaging*. This study shows that the packaging staff members who had undergraduate qualification or above had a lower defect rate in packaging compared with those who did not have an undergraduate qualification. This is consistent with the study of Wu et al. [10] who found that highly educated staff members generally possess solid and comprehensive theoretical knowledge and are more capable of discovering and reporting problems; moreover, they probably had a better understanding of the importance of patient safety culture and exhibited a more rigorous work attitude. Thus, quality of packaging handled by highly educated staff members might be satisfactory, even when there were insufficient staff to handle it. By contrast, staff members with low levels of education might lack professional self-identity, and were more likely to experience job burnout [11], and might be less capable of ensuring that packing met the necessary standards. (3). *Time period of packaging*. The results of this study also show that there were more defects in instrument packaging in the afternoon compared with that in the morning. Staff members tended to be less patient and meticulous when approaching the end of their work shift. Towards the end of the shift, staff members were more likely to rush through their work and pay less attention. Some staff might become drowsy in the afternoon and more likely to make mistakes. Furthermore, instruments from morning operating lists arrived in CSSD in the late morning or early afternoon. and instruments from afternoon operating lists also arrived in CSSD in the afternoon, therefore, there might be an increased workload for afternoon packaging staff members. There were more defects in instrument packaging in the afternoon, which may be also explained by the insufficient afternoon staffing levels. (4) *Instrument structure*. In this study, there was a high percentage of defects in the instruments of a complex structure. It is because dirt and bacteria are more difficult to remove in the instruments of a complex structure. If the instrument cleaning process does not meet the cleaning criteria, i.e. dirt or blood stains remaining on the instruments [12], then the instrument packaging quality in the next step of the packaging process is affected. (5) *Packaging materials*. Packaging materials were used to isolate bacteria inside the package from that outside the package. The usage of instruments taken from packaging materials that are non-intact or unclean can lead to nosocomial infections in patients [13]. Li et al. [13] reported that different types of packaging materials have different degrees of bacterial retention. Our study shows that the efficient group had high wrapping perfection and wrapping cleaning compliance rates, but we did not research the relationship between packaging material types and packaging defects. A further study about it will be needed. (6) *Instrument cleaning*. Surgical instruments become contaminated during operations, and must be thoroughly cleaned, disinfected and sterilized [14]. Due to inadequate implementation of inspection procedure, cleaning for instrument packaging was not always carefully checked, leading to packaging defects.

### Recommended prevention strategies

Online and offline training concerning required methods and skills of surgical instrument packaging, infection, and sterilization is recommended for packaging staff in order to enable them to improve their understanding of packaging/instrument names, purposes, specifications, structures, performance, performance testing methods, standard assembly methods, arrangement order, instrument placement, and significance of surgical instrument packaging [12, 15]. Situational simulation training could enable packaging staff to become familiar with packaging defects prior to inspecting them in practice. It is advisable to place the notices bearing details of packaging standards and clear instructions in conspicuous positions in the workplace for the edification of the packaging staff.

It is also recommended that Surgical Instrument Packaging Reference Drawings be supplied as a standardized reference for packaging procedures. Reference drawings of instruments comprising pictures and texts should detail the shapes, structures, assembly, arrangement order, and instrument placement. The texts should explain the name, model, specification, type, assembly method, arrangement order, and instrument placement, and highlight the instruments that are likely to cause confusion and errors.

Surgical instruments should be maintained prior to packaging. It is necessary to check the cleanliness of the surgical instruments and wrapping, and ensure normal function of instruments and intactness of wrapping. Defective instruments and wrapping need cleaning and replacement before packaging. Instruments should be assembled, arranged, and placed according to the reference drawings, and chemical indicators appropriately assembled. After checking, the package is sealed and labeled. Thereafter, the consistency between labels outside the packages and the instruments inside the packages should be checked [16].

The packaging materials meeting the requirements of surgical instrument packaging criteria are recommended [17]. A non-conformance tracking system is recommended for prompt identification of defects and for determining the causes of packaging defects. It is advisable to create a mentoring system where a senior person and a junior person or a highly educated person and a person with a lower level of education, work as a team. Flexible scheduling is recommended. More senior or highly educated persons need to be added to the staff pool during busier hours.

## Conclusion

In summary, defects in surgical instrument packaging are linked to factors relating to length of service, educational background, time period of packaging, number of staff dealing with packaging, instrument structure, whether the wrap is intact, whether the wrap reaches the required quality of cleaning, and whether the instruments are satisfactorily cleaned in compliance with guidelines. Recommendations for reducing incidences of defects include improved scheduling of packaging workload, greater provision of training in packaging skills, and standardization of packaging procedure, thereby improving surgical instrument packaging quality, reducing potential risk of nosocomial infection, and ensuring patient safety.

## Data Availability

The datasets used and/or analysed during the current study are available from the corresponding author on reasonable request.

## Abbreviations

CSSD: Central sterile supply department
